# ApoE Production in Human Monocytes and Its Regulation by Inflammatory Cytokines

**DOI:** 10.1371/journal.pone.0079908

**Published:** 2013-11-14

**Authors:** Sten Braesch-Andersen, Staffan Paulie, Christian Smedman, Sohel Mia, Makiko Kumagai-Braesch

**Affiliations:** 1 Mabtech, Nacka Strand, Sweden; 2 Mabtech and Center for Molecular Medicine, Infectious Diseases Unit L8:01, Karolinska Institutet, Karolinska University Hospital, Stockholm, Sweden; 3 Applied Immunology, Center for Molecular Medicine, Karolinska University Hospital, Department of Clinical Neuroscience, Karolinska Institutet, Stockholm, Sweden; 4 Mabtech and CLINTEC, Division of Transplantation Surgery, Karolinska Institutet, Stockholm, Sweden; King’s College London School of Medicine, United Kingdom

## Abstract

The apoE production by tissue macrophages is crucial for the prevention of atherosclerosis and the aim of this study was to further elucidate how this apolipoprotein is regulated by cytokines present during inflammation. Here we studied apoE production in peripheral blood mononuclear cells (PBMC) and analysis was made with a newly developed apoE ELISpot assay. In PBMC, apoE secretion was restricted to monocytes with classical (CD14^++^CD16^−^) and intermediate (CD14^+^CD16^+^) monocytes being the main producers. As earlier described for macrophages, production was strongly upregulated by TGF-β and downregulated by bacterial lipopolysaccharide (LPS) and the inflammatory cytokines IFN-γ, TNF-α and IL-1β. We could here show that a similar down-regulatory effect was also observed with the type I interferon, IFN-α, while IL-6, often regarded as one of the more prominent inflammatory cytokines, did not affect TGF-β-induced apoE production. The TNF-α inhibitor Enbrel could partly block the down-regulatory effect of IFN-γ, IFN-α and IL-1β, indicating that inhibition of apoE by these cytokines may be dependent on or synergize with TNF-α. Other cytokines tested, IL-2, IL-4, IL-12, IL-13, IL-17A and IL-23, had no inhibitory effect on apoE production. In contrast to the effect on monocytes, apoE production by primary hepatocytes and the hepatoma cell line HepG2 was more or less unaffected by treatment with cytokines or LPS.

## Introduction

Apolipoprotein E (apoE), a component of HDL and the main lipid transporting protein in the brain, has been shown to have anti-inflammatory, anti-atherogenic and immune modulatory properties [1], [2], [3], [4]. It is a 34 kD glycosylated and sialylated protein [5], [6], [7], [8] prone to form homo- and hetero-dimers [9], [10]. Although most of the apoE found in blood stems from the liver, it is also produced by various cells throughout the body, including astrocytes and macrophages [11]. It has been shown that apoE, produced by macrophages in blood vessel walls, is a critical component in the prevention and healing of atherosclerotic plaques [4], [12], [13], [14] and the regulation of apoE in these cells has become an important area of research. This interest has been further triggered by the recognition of apoE not only acting as a lipid transporter but also as an important immunoregulatory molecule with effects on both T cells and cells of the innate immune system [1], [2], [15], [16], [17], [18].

ApoE production and secretion by macrophages is strongly enhanced after exposure to TGF-β [19], an effect that has been shown to be inhibited by LPS as well as by several pro-inflammatory cytokines including TNF-α IFN-γ and IL-1β [19], [20]. Using apoE-deficient mice, Hayashi et al. have shown that Toll-like receptor 2 (TLR2) is partly responsible for the pathogen-induced inflammatory atherosclerosis through mediating the induction of IFN-γ, IL-1β, IL-6 and TNF-α in the atherosclerotic lesions [21]. Other authors have also shown that IFN-γ, IL-1β, GM-CSF and TNF-α inhibit apoE production in macrophages [19], [22], [23], although there have been conflicting reports on the role of TNF-α [24], [25]. ApoE production in mixed rat glial cell cultures has, on the other hand, been reported to increase by the addition of IL-1β [26]. The role of IL-6 in the induction of inflammatory atherosclerosis seems more complex. Madan et al. have shown that mice lacking IL-6 are more susceptible to atherosclerosis [27]. However, it has also been shown that large injections of IL-6 make atherosclerotic plaques bigger [28].

The aim of this study was to further elucidate the role of cytokine regulation of apoE production and secretion and to test some cytokines not previously used for modulation of apoE production. For the purpose, we used peripheral mononuclear cells (PBMC) and isolated monocytes from healthy volunteers and analysis was performed at the single cell level using a novel apoE ELISpot assay.

## Materials and Methods

### Cells

PBMC were isolated from buffy coats from healthy volunteers (approved by Regionala Etikprövningsnämnden Stockholm, 2006/227-31/1) using Ficoll-Hypaque (GE Healthcare, Uppsala, Sweden) according to the manufacturer’s instructions. If not used immediately, the purified cells were suspended in RPMI (Gibco, BRL, Life Technology Ltd. Paisley, Scotland) supplemented with 10% DMSO (Sigma Aldrich Sweden, Stockholm, Sweden) and 20% fetal calf serum, FCS (HyClone, Thermo Scientific, Logan, UT, USA) and frozen in a Nalgene Cryo 1^o^ freezing container (Nalgene Nunc International, Rochester, NY, USA) before transfer to liquid nitrogen. In some experiments, cells were further separated into a CD14^+^ and a CD14^−^ population using anti-CD14-coupled magnetic beads (CD14 IMAG, BD, San Diego, CA, USA) and following the manufacturer’s instructions.

Fluorescence-activated cell sorting (FACS) of PBMC into classical monocytes (CD14^++^CD16^−^), intermediate monocytes (CD14^++^CD16^+^), non-classical monocytes (CD14^+^CD16^++^) and double negative cells (CD14^−^CD16^−^), was done by the Karolinska core-facility, Huddinge Hospital, on fresh PBMC from blood collected with BD Vacutainer® blood collection tubes (BD Biosciences, Franklin Lakes, NJ, USA) containing heparin. The fluorescent sorter was a FACSAria (BD Biosciences). Phycoerythrin (PE)-conjugated anti-CD14 mAb (clone M5E2) was from BD Biosciences and Alexa Fluor 488-conjugated anti-CD16 mAb (clone 3G8) was purchased from BioLegend (San Diego, CA, USA).

Monocyte-derived macrophages were generated from monocytes freshly isolated from PBMC by use of a CD14^+^ positive selection kit (Miltenyi Biotech, GmbH, Bergish Gladback, Germany) followed by culturing in RPMI 1640 supplemented with 10% FCS (Sigma, St. Louise, MO, USA), 50 ng/ml M-CSF, 100 U/ml penicillin, 100 µg/ml streptomycin, 2 mM L-glutamine (all from Gibco) in 6-well plates and at a concentration of 2×10^6^ cells/well. Medium was replaced on the 3rd and 5th day with M-CSF-containing medium and cells were harvested on day 7.

HepG2 cells were purchased from ATCC (Rockville, MD, USA) and human hepatocytes were a kind gift from Dr. Ewa Ellis, Karolinska Institutet, Stockholm, Sweden (approved by Regionala Etikprövningsnämnden Stockholm, 2010/678-31/3). Cells were maintained as adherent cultures in DMEM medium (Gibco) with 10% FCS and were detached by treatment with trypsin/EDTA (Gibco) before used.

### Cytokines and other reagents

The cytokines IL-1β, IL-2, IL-4, IL-6, IL-12, IL-17A, IL-23, M-CSF and GM-CSF were all from Peprotech Inc while TNF-α, IFN-γ and IL-13 were from both Bender MedSystems (Vienna, Austria) and Peprotech Inc. IFN-α (Multipheron^®^) was produced by Swedish Orphan Biovitrum Sverige AB (Umeå, Sweden) and TGF-β was from R&D Systems (Minneapolis, MN, USA). The TNF-α antagonist Enbrel was from Pfizer (New York, NY, USA). Lipopolysaccharide (L2654, Escherichia coli 026:B6) and Polymyxin B were both from Sigma Aldrich and mouse monoclonal anti-human CD3 and CEF (pool of 23 antigenic peptides from Cytomegalovirus, Epstein-Barr virus and Flu virus) were from Mabtech AB (Nacka Strand, Sweden). Suitable reagent concentrations were defined by titration in relevant systems. TGF-β,TNF-α, IFN-γ, IL-1β, IFN-α, IL-2, IL-4, IL-6, IL-12, IL-13. IL-17A and IL-23 were all used at 10 ng/ml, unless stated differently in the figure legend. M-CSF used for the generation of monocyte-derived macrophages was added at 50 ng/ml. The TNF-α antagonist, Enbrel was used at 50 µg /ml, Polymyxin B at 10 µg/ml and LPS at 0.1, 1 or 100 ng/ml. For stimulation of T cells, anti-CD3 was added at 100 ng/ml and CEF at 2 µg/ml of each peptide.

### ApoE ELISpot, FluoroSpot and ELISA

The ELISpot assay was performed using apoE-specific mouse monoclonal capture (mAb E276) and detection (mAb E887-biotin) antibodies (Mabtech) and following the conditions recommended by the manufacturer. In short, PVDF-backed 96-well filter plates (MSIPS4510) from Millipore (Billerica, MA, USA) were pre-treated with ethanol and the capture antibody, mAb E276 was added at 15 µg/ml (100 µl/well) and allowed to bind for at least four hours at room temperature or at 4°C overnight. The plates were washed three times in sterile PBS to remove unbound antibodies and blocked with cell culture medium (RPMI with 10% FetalClone 1) for one hour. Cells, with and without the substances to be tested, were added in a total volume of 150 µl/well and left to incubate for approximately 20 hours at 37°C in a humidified 5% CO_2_-supplemented incubator. After incubation, cells were removed by washing with PBS in an ELISA washer (ELx50, BioTek Instruments, Winooski, VT, USA) followed by addition and incubation with biotinylated detection antibody (mAb E887-biotin, 1 µg /ml) at room temperature for 1 hour. After further washing and incubation for 1 hour with Streptavidin-ALP (Mabtech), spots were visualized by the addition of a precipitating substrate, BCIP/NBT (Mabtech) and evaluation and counting of spots were done using an ELISpot Reader (AID, Strassberg, Germany).

For the adherent cells (HepG2 and hepatocytes), a slightly modified protocol was used; after overnight culture and the subsequent washing with PBS in the ELISA washer, 100 µl/well of PBS supplemented with 1 mM EDTA was added and left to incubate for 10 minutes at 37°C. This way, cells were detached and could be removed by a subsequent wash with PBS. After this step, the procedure continued with addition of biotinylated detection mAb as described above.

FluoroSpot (Mabtech) was performed essentially as previously described [29] and using similar conditions as for the ELISpot. However, to minimize background fluorescence, 96-well plates with a low fluorescent PVDF membrane were used and plates were coated with two capture antibodies instead of one. Similarly, detection was made with two detection antibodies, one conjugated with a peptide tag (anti-TNF-α) and the other with biotin (anti-apoE and anti-IL-6). Finally, detection was made by the addition of a FITC-labeled anti-tag mAb and Streptavidin conjugated with Cy3 followed by a short incubation with a fluorescence enhancer. Analysis and counting of spots were made in an ELISpot/FluoroSpot reader system (iSpot, AID, Strassberg, Germany) where fluorescent spots were counted utilizing separate filters for FITC and Cy3.

ApoE concentrations in cell supernatants were measured by ApoE ELISA kit (Mabtech) following the manufacturer’s instructions.

### Statistical analysis

Results shown are in the form of means and standard deviation. Statistical analysis was performed with the Graphpad Prism 6 program. For analysis the Mann-Whytney-U-test was used. The differences were considered as statistically significant if values were *p* < 0.05.

## Results

### Monocytes are the main producers of apoE in PBMC

**Figure 1:**
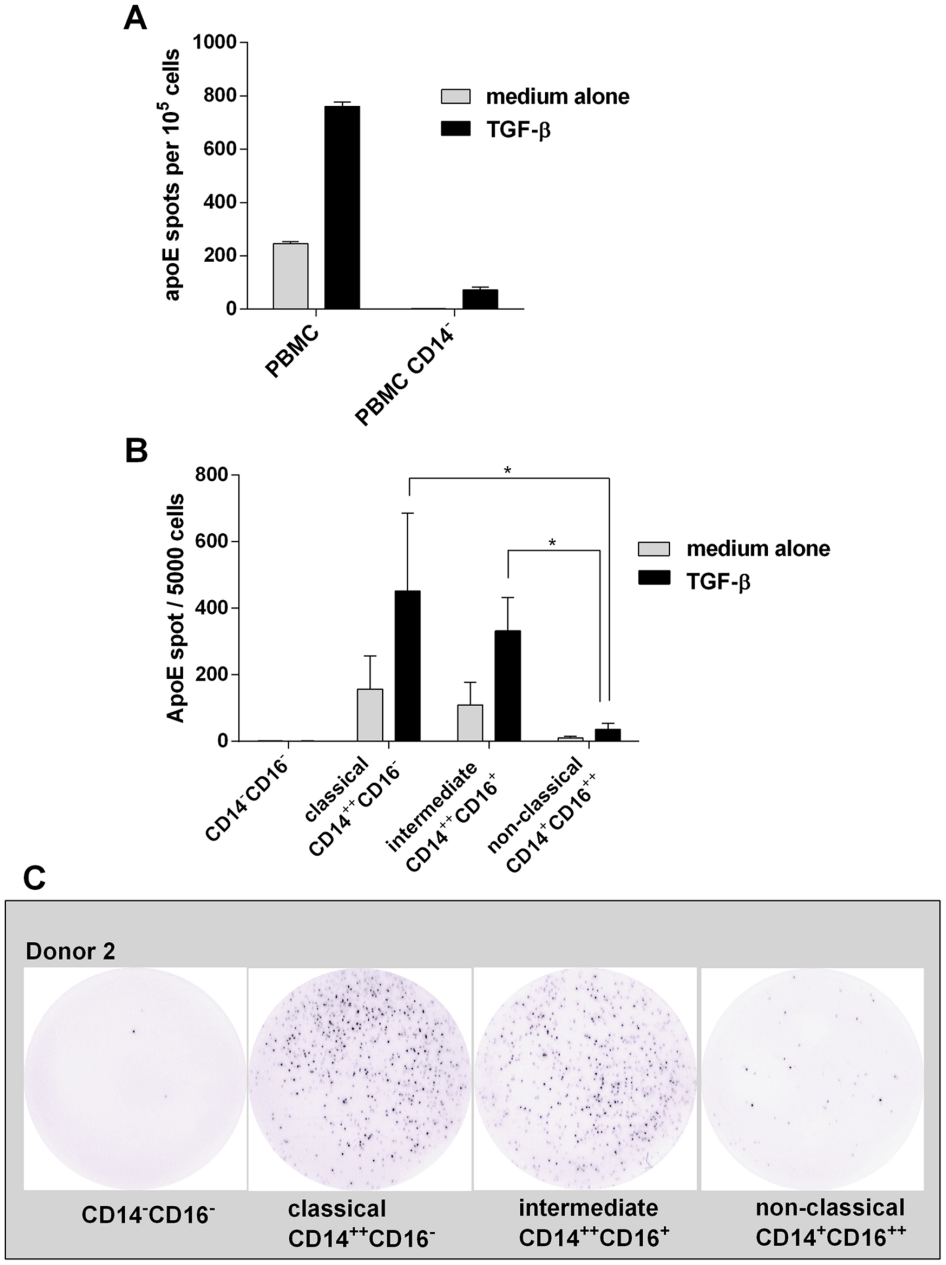
ELISpot analysis of ApoE secretion in response to TGF-β -treatment. **A**) Whole PBMC were incubated overnight in the presence (black bars) or absence (grey bars) of TGF-β (10 ng/ml) and the number of apoE-secreting cells was determined by ELISpot. Comparison was made with PBMC depleted of CD14^+^ cells. Results shown are from one representative donor (means ± SD of quadruplicates). Similar results were obtained in three separate experiments with three different donors. **B**) PBMC from three donors were sorted into different monocyte subpopulations and non-monocytic cells by a fluorescence-activated cells sorter and tested in ELISpot for apoE production using 5×10^3^ cells/well. Values represent means ± SD of three donors. * p<0.05. Individual data are shown in supplementary figure 1. **C**) Representative examples of apoE spots (5×10^3^ cells/well) produced by non-monocytic cells, classical monocytes, intermediate monocytes and non-classical monocytes in response to TGF-β.

Macrophages have previously been shown to produce apoE in response to TGF-β. To investigate whether TGF-β also induced apoE secretion in peripheral blood cells, PBMC were cultured with or without bioactive TGF-β for 20 hours and analysed in the ELISpot assay. As shown in figure 1A, a limited number of cells were seen to spontaneously produce apoE, a number that was increased severalfold in the presence of TGF-β. By depleting CD14^+^ monocytes from PBMC we could further demonstrate that apoE secretion was mainly confined to the monocyte population (Fig. 1A). Given the monocyte origin of apoE, we also sorted cells into the three generally accepted subpopulations of classical, non-classical and intermediate monocytes and investigated their apoE-secreting capacity. For this purpose, freshly prepared PBMC were sorted by FACS into CD14^++^ CD16^−^ (classical), CD14^++^ CD16^+^ (intermediate) and CD14^+^ CD16^++^ (non-classical) as well as CD14^−^CD16^−^ cells (primarily lymphocytes). The different populations were incubated with and without TGF-β. As seen in figures 1B (individual data Fig. S1) and 1C the number of secreting cells was highest in the classical monocytes with approximately 10% positive cells in the three donors tested. Slightly less secreting cells were observed in the intermediate population and very few in the non-classical population. No or almost no detectable production was seen in the remaining population of non-monocytic cells.

### Inhibition of apoE secretion by pro-inflammatory cytokines – a key role for TNF-α

**Figure 2:**
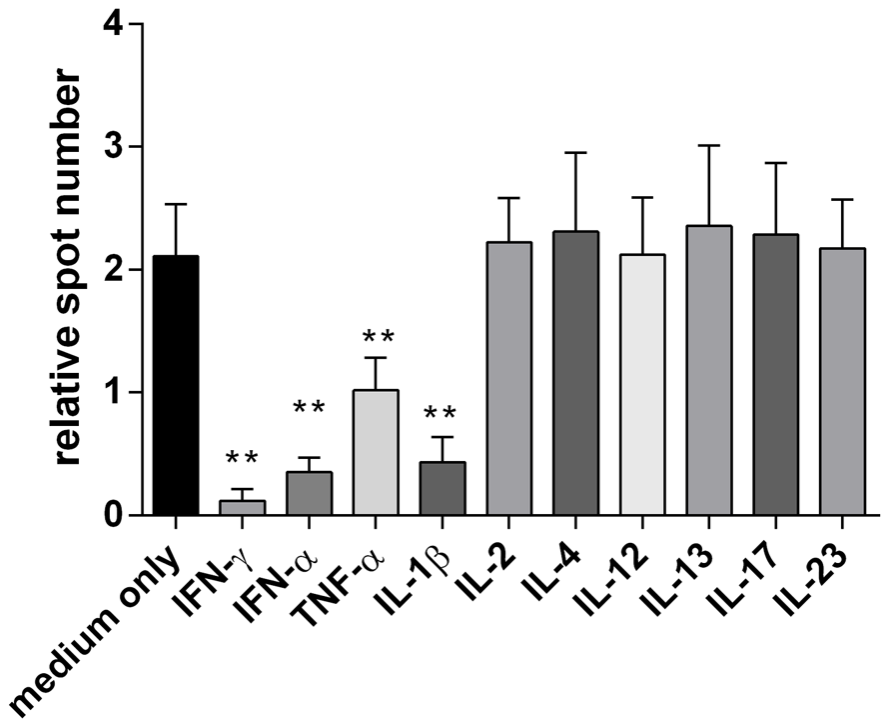
Cytokine effect on TGF-β-induced apoE production. PBMC (100×10^3^ cells/well) in medium containing 10 ng/ml TGF-β ( =  medium only) were incubated for 20 hours together with different cytokines. All cytokines were used at 10 ng/ml. Results are depicted as relative spot numbers obtained by dividing the number of apoE spots in cytokine-containing wells with the number of spots in wells with cells in medium without TGF-β. Tests were performed with 8 donors and results are shown as means ± SD, ** p<0.01.

In figure 2, a number of cytokines, known to be involved in the regulation of immunocompetent cells, were tested for their potential effects on apoE production in PBMC. In accordance with previous observations on macrophages, the pro-inflammatory cytokines TNF-α, IFN-γ and IL-1β effectively inhibited both spontaneous and TGF-β induced apoE secretion and, as demonstrated here for the first time, so did IFNα (Fig. 2). In contrast, IL-2, IL-17A, IL-23 and the two Th2 associated cytokines IL-4 and IL-13 had no negative effect on apoE production. Furthermore, IL-4 and 13, enhanced apoE production by unstimulated PBMC to some extent (Fig. S2).

**Figure 3:**
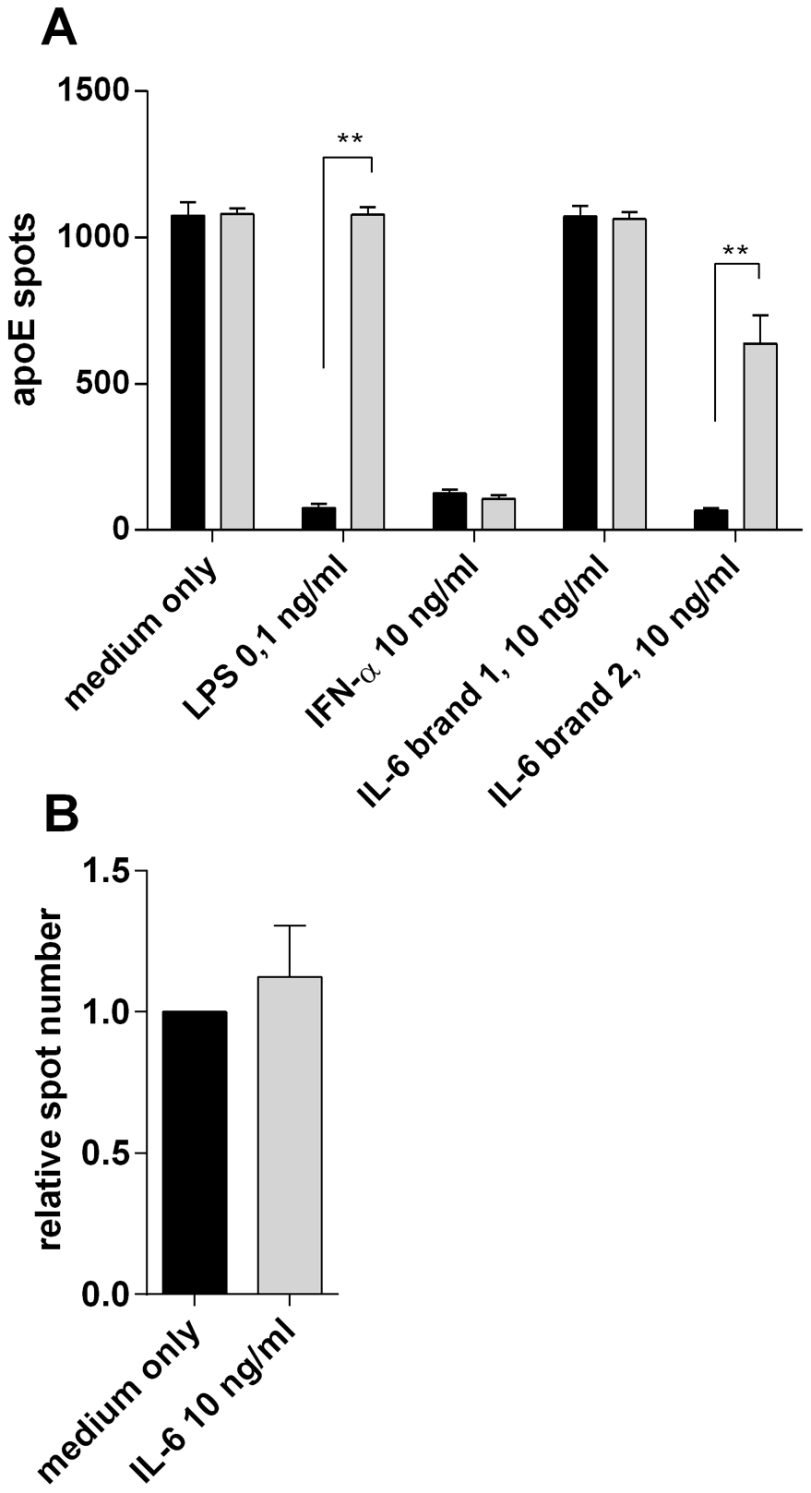
Verified lack of IL-6 effect on apoE production in the presence of the LPS-inhibitor Polymyxin B. **A**) Example of how contaminating LPS in cytokine preparations can affect apoE production. TGF-β-treated PBMC (100×10^3^ cells/well) were incubated for 20 hours in the presence of LPS (0.1 ng/ml), IFN-α, or either of two preparations of IL-6 (brand 1 and 2). All cytokines were used at 10 ng/ml. Cells were incubated with (grey bars) or without (black bars) Polymyxin B (10 µg/ml). Results shown are from one representative donor (means ± SD of triplicate). (**B**) PBMC (100×10^3^ cells/well) were incubated for 20 hours in the presence of TGF-β (10 ng/ml) and Polymyxin B (10 µg/ml) ( =  medium only) with or without IL-6 (10 ng/ml). Relative spot numbers for each donor were calculated by dividing apoE spot numbers of IL-6 treated PBMC with the number of spots obtained without IL-6. Results shown are means ± SD of 4 donors.

In some experiments, we observed discordant results when using IL-6 from two different vendors, with one preparation inhibiting apoE secretion and the other not. Considering the strong inhibitory effect seen with LPS and the fact that recombinant cytokines may sometimes contain trace amounts of contaminating LPS, the two IL-6 preparations were tested in the presence and absence of Polymyxin B (Fig. 3A). This substance is a potent inhibitor of LPS activity and, in line with this, it effectively blocked the inhibitory effect of LPS while not affecting the inhibition of apoE secretion seen with IFN-α (Fig 3A). However, in the case of IL-6, it significantly reduced the inhibition observed with one of the IL-6 preparations. Taken these results into account, all cytokines displaying inhibitory effects were carefully retested to confirm that their effects were not due to LPS contamination. Furthermore, we could confirm that IL-6, albeit normally considered proinflammatory, did not inhibit apoE secretion (Fig 3B).

**Figure 4:**
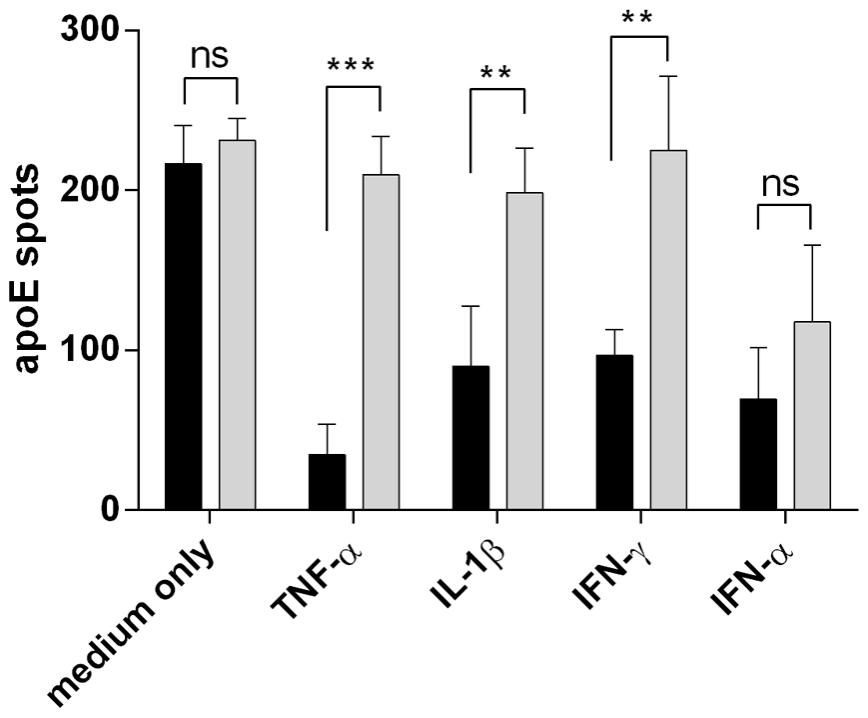
The role of TNF-α and the TNF-α antagonist, Enbrel, in regulating apoE production. CD14^+^ cells (30×10^3^ cells/well), positively selected from PBMC, were incubated for 20 hours in ELISpot plates in the presence of TGF-β (10 ng/ml) and Polymyxin B (10 µg/ml) with or without Enbrel (50 µg/ml). Black bars indicate apoE spot number without Enbrel and grey bars with Enbrel. Inflammatory cytokines were added at 10 ng/ml. Results shown are means ± SD of 4 donors. *** p<0.001, **p<0.01, ns: not significant.

The inhibitory effect of IL-1β and IFN-γ was also observed when testing purified monocytes (Fig. 4). As expected, TNF-α, previously shown to inhibit apoE in macrophages (19), led to a similarly reduced production as did IFN-α, a cytokine normally induced by viral infection. Interestingly, addition of the TNF-α antagonist, Enbrel, was shown to interfere with the inhibitory effect of not only TNF-α but also of IFN-γ and IL-1β.). This suggests that inhibition of apoE with these inflammatory cytokines may either work via induction of TNF-α or by acting in a synergistic manner to enforce the TNF-α signal.Enbrel also appeared to consistently affect the apoE inhibition seen with IFN-α although the difference was not statistically significant. (p =  0.171)

### T cell suppression of apoE production

**Figure 5:**
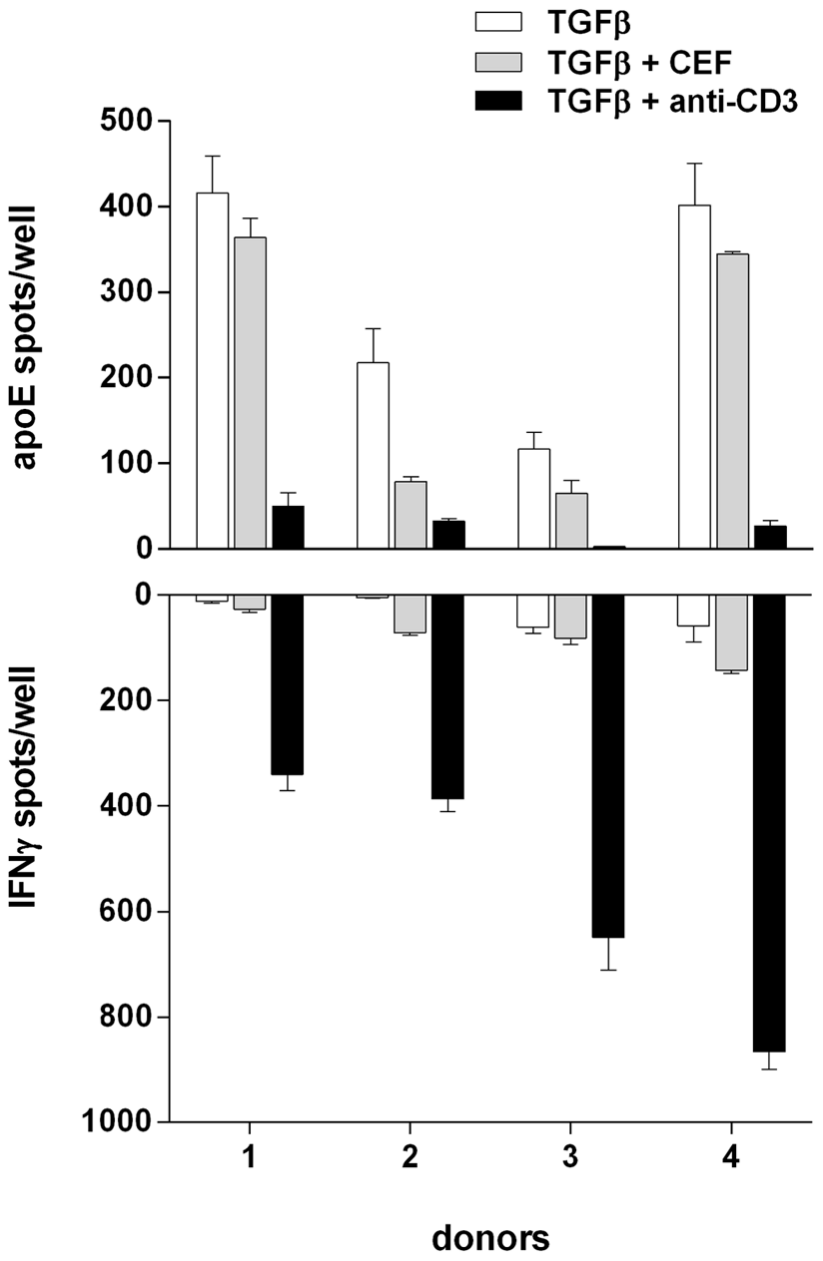
Inhibition of ApoE secretion by PBMC in the presence of activated T cells. PBMC (100×10^3^ cells/well) from 4 different donors were incubated for 20 hours in the presence of TGF-β (10 ng/ml) and Polymyxin B (10 µg/ml) with or without anti-CD3 (100 ng/ml) or CEF (2 µg/ml) and analysed for secretion of apoE (top) and IFN-γ (bottom) in the ELISpot assay. Values represent means ± SD of triplicates.

While apoE is believed to play an important protective role in atherosclerosis, the reverse is true for T cells [30], [31]. Through their secretion of cytokines such as IFN-γ, activated T cells in atherosclerotic plaques may contribute to the inflammatory state that is characteristic of these lesions. To assess the potential interrelationship between monocytes and T cells, PBMC were simultaneously stimulated with TGF-β together with either anti-CD3 or a pool of viral antigenic peptides (CEF) to which most people have a T-cell response. As seen in figure 5, cultures in which T cells had been polyclonally activated showed strong IFN-γ responses and a more or less complete lack of apoE-secreting cells while the antigen-specific and lower IFN-γ responses resulted in a more moderate inhibition of apoE secretion.

### ApoE production in macrophages

**Figure 6:**
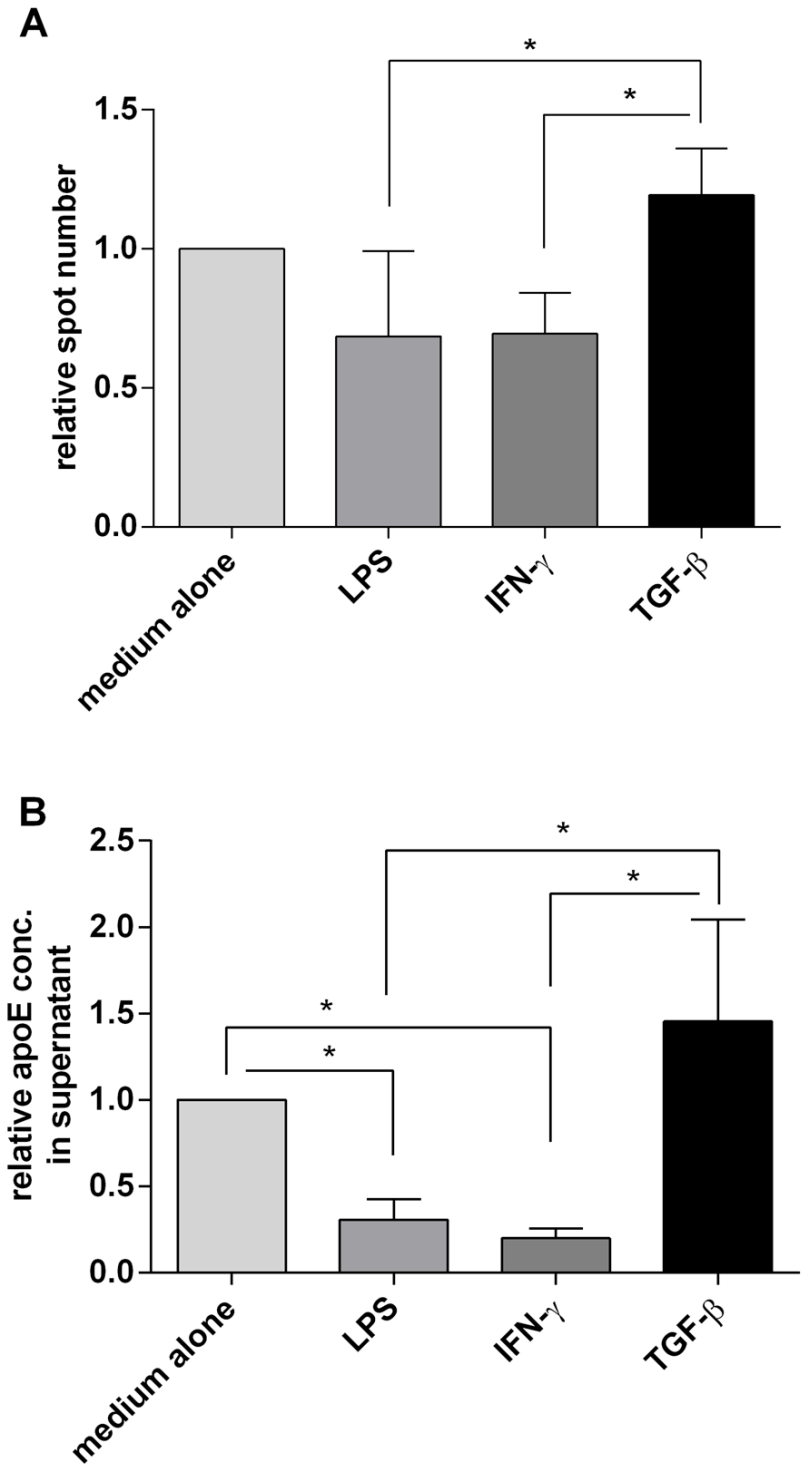
ELISpot and ELISA analysis of apoE secretion by cultured macrophages. **A**) ApoE secreting cells were measured by ELISpot using monocyte-derived macrophages (800 cells/well) incubated for 18 hours in medium alone or in the presence of LPS (1 ng/ml), IFN-γ (10 ng/ml) or TGF-β (10 ng/ml). Results are shown as relative spot numbers where spots in wells with only medium is set to 1. **B**) ELISA analysis of ApoE content in culture supernatants of monocyte-derived macrophages (7000 cells/well) incubated for 48 hours with the same substances as in A. As above, results are given as relative ELISA values where the concentration of ApoE in wells with only medium were set to 1. Figures represent means ± SD of four donors, * p<0.05.

Given that earlier reports of apoE secretion by immune cells have relied primarily on work performed with macrophages, we wanted to compare how apoE production in macrophages compared to that observed in blood monocytes. As expected and here demonstrated with both ELISpot and ELISA (Fig. 6) macrophages, generated by a 7-day propagation of isolated CD14^+^ cells in M-CSF, secreted significant amounts of apoE. This production could be upregulated by TGF-β and downregulated by LPS and IFN-γ. However, compared to monocytes the effects on macrophages were modest and more clearly discerned with ELISA than ELISpot.

**Figure 7:**
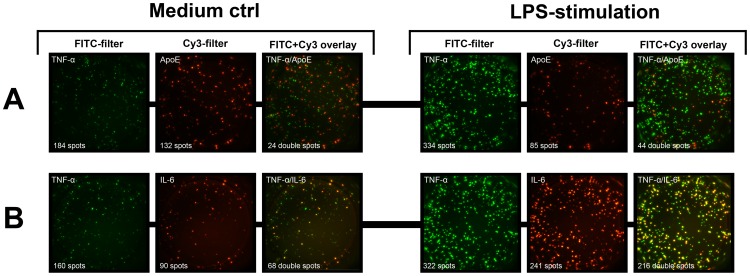
Fluorospot analysis of apoE, TNF-α and IL-6 secretion by macrophages. Monocyte-derived macrophages (800 cells/well) were incubated 20 hours in the presence or absence of LPS (100 ng/ml) and cells secreting apoE, TNF-α and IL-6 were determined in the FluoroSpot assay. The figure shows examples of Fluorospot wells with the simultaneous detection of apoE (red spots) and TNF-α (green spots) (**A**), as well as IL-6 (red spots) in combination with TNF-α (green spots) (**B**). The numbers of double producing cells were determined as spots having the same centre point in an image overlay of FITC and Cy3 images. Similar results were obtained using four different donors. More data are shown in Table S1.

As macrophages spontaneously produce not only apoE but also cytokines such as IL-6 and TNF-α, we conducted some preliminary experiments looking at to what extent these were produced by the same cells or not. For this purpose we used the FluoroSpot technique, a variant of the ELISpot which by employing fluorescent detection allows simultaneous detection of more than one secreted product. As shown in figure 7 (left part) with macrophages incubated overnight in only medium, there were a similar number of cells secreting apoE and TNF-α. However, the two proteins were for the most part produced by separate cells with less than 20% of the apoE-producing cells also secreting TNF-α. In comparison, more than 70% of cells secreting the cytokine IL-6 also secreted TNF-α. As expected, addition of LPS to the cultures (Fig. 7, right part) resulted in a reduced number of apoE-secreting cells whereas IL-6 and TNF-α were both strongly upregulated. However, concomitant with the reduced number of cells secreting apoE, the proportion of these cells also secreting TNF-α increased from 20 to about 50%. Four donors were tested and individual data are shown in Table S1.

### ApoE production in hepatocytes

As most apoE present in plasma has been reported to come from the liver, we investigated whether the same cytokines that inhibited apoE production in monocytes had a similar regulatory effect on liver cells. To address this, we used the hepatoma cell line HepG2 as well as freshly isolated hepatocytes. As seen in figure S3A, HepG2 cells showed a spontaneous secretion of apoE that was not enhanced by TGF-β and was also unaffected by the addition of pro-inflammatory cytokines as well as LPS. The same was true for normal hepatocytes (data not shown). However, when supernatants were analysed by ELISA a slight, but significant, inhibition could be seen after adding IFN-α and TNF-α (Fig. S3B). The ELISA in the figure was done on 20-hour supernatants, but similar results were observed with supernatants collected after 48 hours.

### ELISpot vs. ELISA

From the experiments with macrophages and HepG 2 cells (Fig. 6 and S3), ELISA could appear as the more sensitive of the two assays with a better capacity to detect minor differences in the amount of secreted apoE, differences that were not as easily discernible at the cellular level.

**Figure 8:**
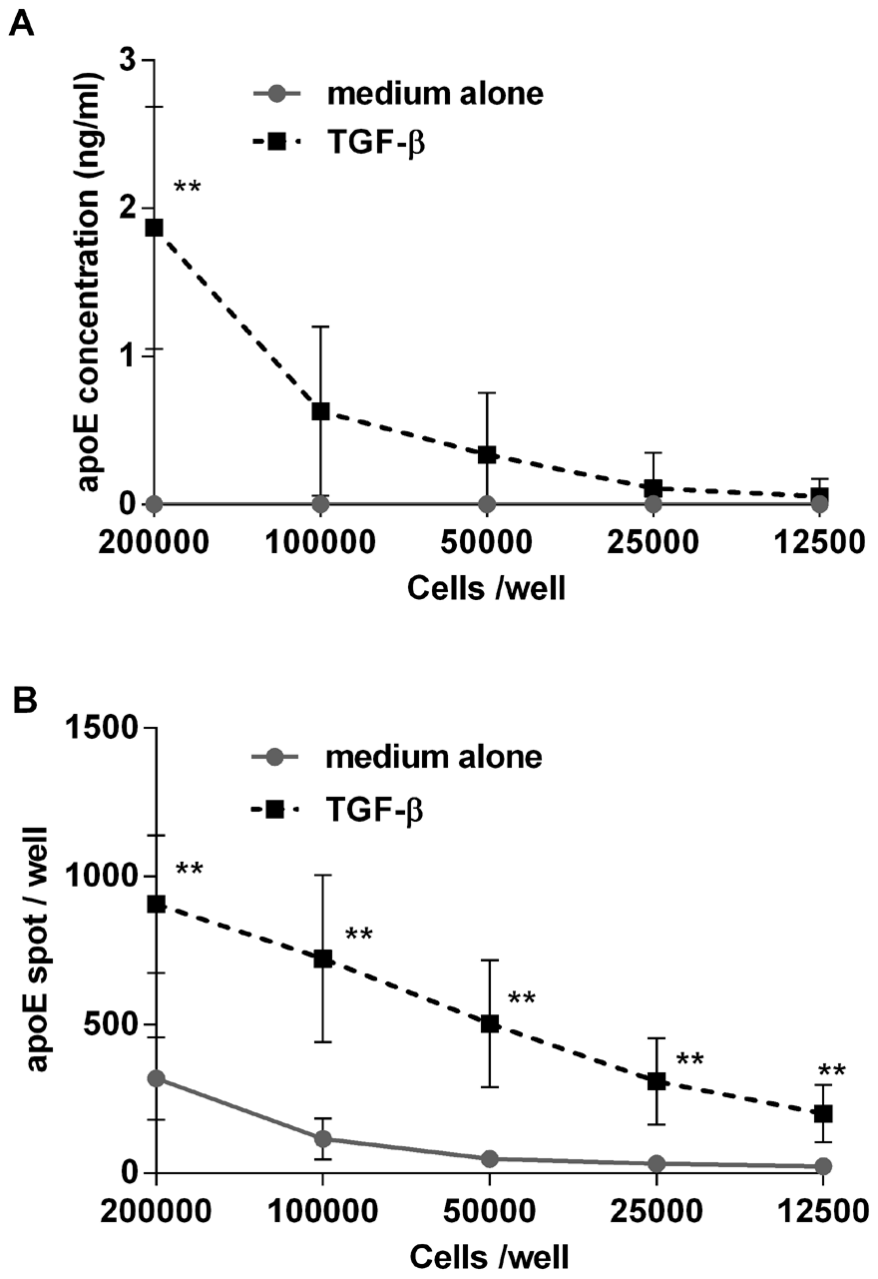
Comparison of apoE measurements by ELISA and ELISpot. PBMC in different cell numbers were incubated for 24-β (10 ng/ml) and secretion of apoE was measured by ELISA (**A**) and ELISpot (**B**). Data represent means ± SD of five donors. Comparison was made between cultures with the same cell concentrations with and without TGF-β. ** p<0.01

To demonstrate the pros and cons of the two assays, different numbers of PBMC were stimulated with TGF-β and the number of secreting cells was determined in ELISpot while, in parallel cell cultures, the amount of apoE in cell supernatants was determined by ELISA. As shown in figure 8, apoE could be measured by ELISA with confidence at cell concentrations of 100×10^3^ cells per well and above whereas in the ELISpot, secreting cells were readily detectable also at the lowest cell concentration of 12.5×10^3^ cells per well. This indicates a generally greater sensitivity of the ELISpot assay. However, the difference in apoE secretion between TGF-β-stimulated and unstimulated cells were more prominent in the ELISA (Fig. 8A). Together the two assays also demonstrate that the TGF-β-related increase in apoE production is likely the result of not only an increase in the number of secreting cells but also an enhanced production by already producing cells.

## Discussion

Inflammation has been identified as the underlying cause of atherosclerosis and a low-grade chronic inflammatory response in the arterial walls has been suggested to drive both the induction and progression of the disease [32]. A better understanding of the mechanisms that cause inflammation in this setting is therefore important and may lay the grounds for new therapeutic approaches.

Contrary to the pro-inflammatory cytokines, which play a pivotal role in inducing and maintaining inflammation, macrophage-produced apoE has been shown to play an equally important but protective role in atherosclerosis [4], [12], [13]. Given its anti-inflammatory properties [3], [33], we have here investigated how production and secretion of apoE is influenced by a number of cytokines, all of which may be present in atherosclerotic lesions. For the purpose, we used a newly developed ELISpot assay by which apoE secretion may be studied at the single cell level.

Using this approach we could demonstrate a low but significant number of apoE-secreting cells in non-activated PBMC, which, similar to what has been observed earlier with macrophages, could be significantly enhanced in the presence of TGF-β. Secretion was essentially limited to monocytes and tests with isolated monocyte subpopulations defined classical and intermediate monocytes as the main producers with few positive cells in the non-classical population. This is interesting not least in view of previous difficulties in distinguishing these subpopulations based on functional characteristics such as the secretion of cytokines [34], [35], [36]. Non-classical monocytes have also been claimed to be the most mature form of monocytes and the most likely to leave the blood stream and entering into tissue [35]. If correct, this suggests that the majority of extravasating monocytes have a limited capacity to produce and secrete apoE.

As most previous knowledge about apoE secretion by immune cells is based on experiences from macrophage cultures, we also used the ELISpot assay to compare apoE secretion in monocytes and macrophages. As evident from this comparison, many of the features seen with monocytes could also be reproduced in macrophages including the upregulatory effect of TGF-β and the corresponding downregulation with IFN-γ and LPS. However, due to the activated state of these cells with an already existing secretion of apoE, the effects were not as distinct and readily detected as in monocytes. Therefore, we think that monocytes may not only provide a more accessible source of cells but that they are also more susceptible to different interventions.

ApoE production in monocytes was inhibited by the pro-inflammatory cytokines IFN-γ, TNFα and IL-1β. This shows that cytokines, typically produced by monocytes and macrophages in response to TLR signalling (TNF-α, IL-1β) or by T cells in response to antigen (IFN-γ, TNF-α), have a direct negative effect on apoE production. Not previously demonstrated, we also observed a similar negative regulation of apoE production with IFN-α This cytokine is produced primarily by plasmacytoid dendritic cells in response to viral RNA and DNA via TLR-7 and -9 [37], [38], [39] and our finding may help to explain the association between some viral infections and an increased risk of atherosclerosis. In addition, there may exist a similar causal relationship between the increased IFN-α levels seen in SLE patients and the higher incidence of atherosclerosis in these patients [40].

Interestingly, IL-6, normally considered a prominent inflammatory cytokine, did not downregulate apoE production. IL-6 has several unique features that set it aside from other inflammatory cytokines [41]. For instance, it is produced in large amounts by muscle cells upon strenuous exercise and it stimulates whole body glucose and lipid metabolism, observations that may underlie the beneficial effects of physical exercise [42], [43], [44]. It has also been shown to alleviate the symptoms of metabolic syndrome [45] and Madan et al [27] have reported that IL-6 protects against atherosclerosis in apoE^−/−^ mice. The many roles of IL-6 have been further discussed by Fisman and Tenenbaum [46], but in line with our observation, more and more data seem to question its role as solely a pro-inflammatory cytokine.

When testing IL-6, we also initially obtained different results when using recombinant IL-6 from two different sources, one showing inhibition the other not. However, the inhibitory effect was reversed when adding polymyxin B, an antibiotic that efficiently binds to and neutralizes LPS. By including polymyxin B in our cultures we could exclude that the downregulation of apoE displayed by the other cytokines was due to contaminating LPS. However, we have subsequently encountered similar problems with other cytokines. This suggests that the phenomenon is not uncommon and indicates the need for caution whenever using recombinant cytokines and maybe particularly when testing their effects on innate immune cells designed to respond to very low levels of bacterial components and other TLR agonists.

With activated T cells frequently present and contributing to inflammation in atherosclerotic plaques, we also looked at apoE secretion in PBMC when simultaneously stimulating T cells either polyclonally via CD3 or with specific antigens. With both types of stimuli, we observed a significant decrease in the number of apoE-secreting cells. Inhibition is here likely dependent on IFN-γ and/or TNFα, both of which are produced by activated Th1 and cytotoxic T cells. Interestingly, in mice, apoE has been shown to inhibit secretion of the Th1-promoting cytokine IL-12 [3], thus indicating that there may exist an intricate balance between apoE-secreting monocytes/macrophages on one hand and IFN-γ/TNF-α-secreting T cells on the other.

By use of the TNF-α inhibitor Enbrel, we could also identify TNF-α as the likely key factor in suppressing apoE production with neutralization of TNF-α not only affecting TNF-α-mediated inhibition but also that of IFN-γ IL-1β and to a lesser extent IFN-α. This shows that the effect of these latter cytokines may either be indirect and work through the induction of TNF-α or they may act synergistically with TNF-α. Already in 1994, Zuckerman and co-workers showed that antibodies to TNF-α prevented the reduction of apoE in mouse macrophages treated with LPS or GM-CSF [25]. They further showed that antibodies to IL-1α, IL-1β and IFN-γ did not have the same effect. The fact that the results with TNF-α neutralization could here be reproduced with human cells using a therapeutically approved TNF-α blocker makes this approach amenable for testing also in humans.

Interestingly, of the cytokines tested and shown to modulate apoE production, TNF-α is the only one produced by both T cells and monocytes. This suggests that TNF-α neutralization could be anticipated to prevent inflammation independent of whether it is caused by an overactivation of T cells or innate immune cells or both.

As an attractive alternative to inhibiting TNF-α, therapy could potentially also be based on an upregulation of apoE production. The identification of factors which can modulate apoE in monocytes and macrophages would therefore be of great interest. If existing, such factor/s could help to change the balance towards a more anti-inflammatory state that may have a beneficial effect not only on atherosclerosis but also on several other conditions characterized by excessive inflammation.

In contrast to monocytes and macrophages, we found that apoE secretion by hepatocytes was only marginally affected by inflammatory cytokines. The effect, which was only seen in ELISA, was manifested as a small but significant decrease of apoE in supernatants from HepgG2 cells after treatment with IFN-α or TNF-α but not IFN-γ or LPS. Similar effects of TNF-α and also IL-1β on HepG2 cells have previously been reported by Song and et al who also, in concordance with our results on monocytes, did not see any inhibition with IL-6 [47]. The fact that we could not observe the same effect in the ELISpot as in ELISA, demonstrates the differences in the two techniques with ELISpot providing the frequency of secreting cells, but with limited information about the amount produced by each cells whereas ELISA gives the concentration of a secreted analyte produced by a collective of cells.

In conclusion, we have demonstrated that apoE ELISpot analysis of PBMC (or purified monocytes) may provide a convenient model to investigate apoE production and regulation. Using this approach, we could confirm the suppressive effect of several known pro-inflammatory cytokines. In addition, we identified IFN-α as a potent inhibitor of apoE production whereas IL-6, generally considered as pro-inflammatory, had no such effect. Providing further support for a causal relationship between activated T cells and the induction of atherosclerosis we could also show a clear reduction in apoE secretion in PBMC containing stimulated T cells. The observations made in this study support that apoE, having a central role as an anti-inflammatory molecule, constitutes a potential therapeutic target in atherosclerosis and other inflammatory-related diseases.

## Supporting Information

Figure S1 **ELISpot analysis of ApoE secretion by different subsets of monocytes in response to TGF-β -treatment.** PBMC from three individual donors were sorted into different monocyte subpopulations and non-monocytic cells by a fluorescence-activated cells sorter and tested in ELISpot for apoE production using 5×10^3^ cells/well. Values represent means ± SD of triplicates.
(TIF)

Figure S2 **Cytokine effect on spontaneous apoE production.** PBMC (100×10^3^ cells/well) were incubated for 20 hours together with different cytokines. All cytokines were used at 10 ng/ml. Results shown are means ± SD using 6 donors. Relative spot numbers were calculated by dividing the number spots in the cytokine containing wells with the number of spots obtained with cells cultured in medium alone. * p<0.05, compared with medium alone.
(TIF)

Figure S3 **ELISpot and ELISA analysis of apoE secretion by cytokine-treated HepG2 cells.** HepG2 cells (2000 cells/well) were incubated for 20 hours with the indicated cytokines (10 ng/ml) or LPS (1 ng/ml). A) The number of apoE secreting HepG2 cells was evaluated using ELISpot. B) ApoE concentrations were measured in cell culture supernatants by ELISA. Values represent means ± SD of sextuplicates. Differences were considered significant for p<0.05 (*).
(TIF)

Table S1 **ApoE, TNF-α and IL-6 fluorospot numbers by monocyte derived macrophages.** 800 macrophages /well were incubated 24 hours with medium only, LPS (100 ng/ml), IFN-γ (10 ng/ml) or TGF-β (10 ng/ml).
(XLSX)
