# Supplementary figures and images for: ApoE Production in Human Monocytes and Its Regulation by Inflammatory Cytokines
Source: PLoS One. 2013 Nov 14;8(11):e79908. doi: 10.1371/journal.pone.0079908 (PMC3828220; doi:10.1371/journal.pone.0079908)

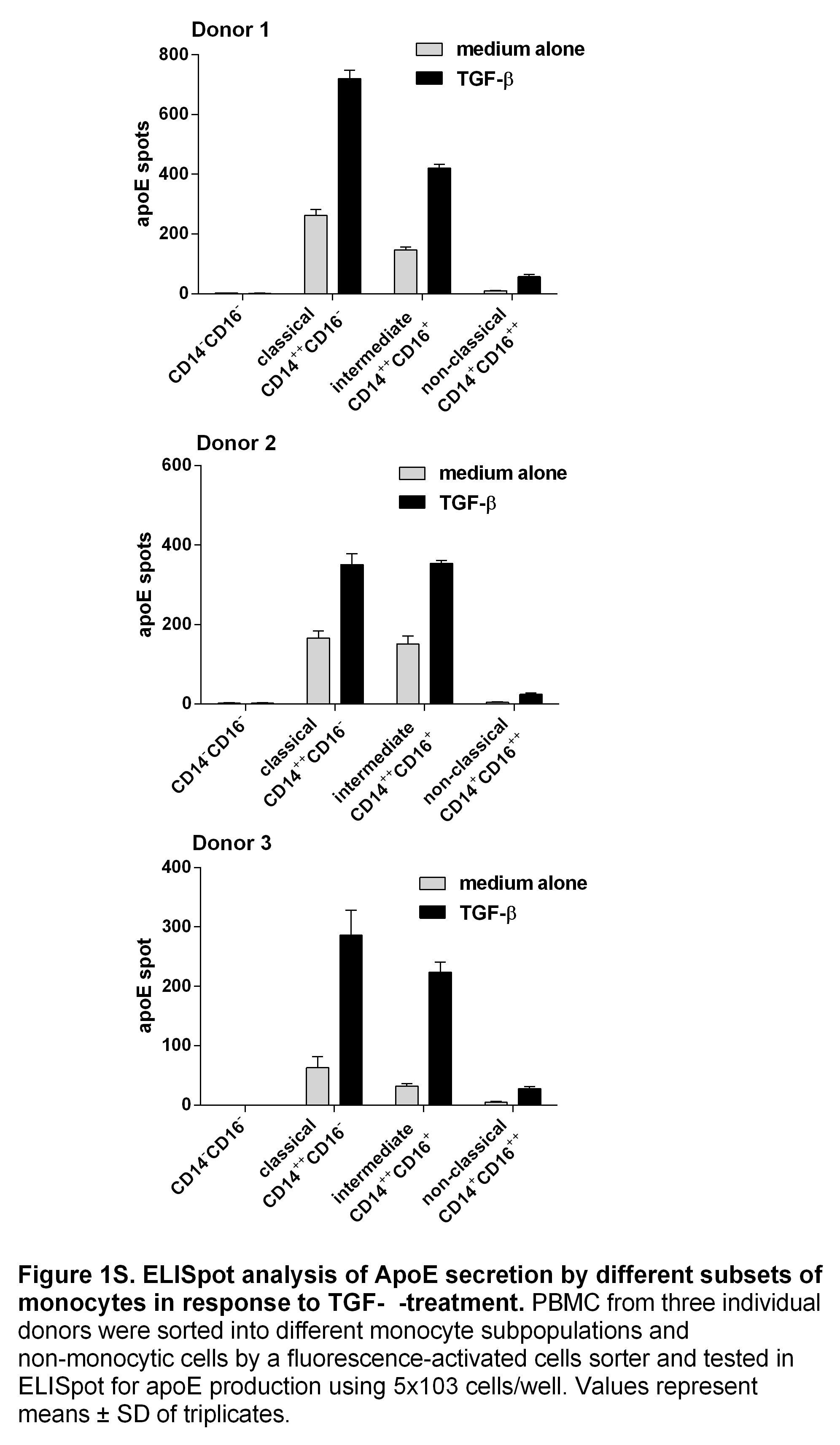

Supplement: Figure S1 — ELISpot analysis of ApoE secretion by different subsets of monocytes in response to TGF-β -treatment. PBMC from three individual donors were sorted into different monocyte subpopulations and non-monocytic cells by a fluorescence-activated cells sorter and tested in ELISpot for apoE production using 5×10^3^ cells/well. Values represent means ± SD of triplicates. (TIF) [file pone.0079908.s001.tif]

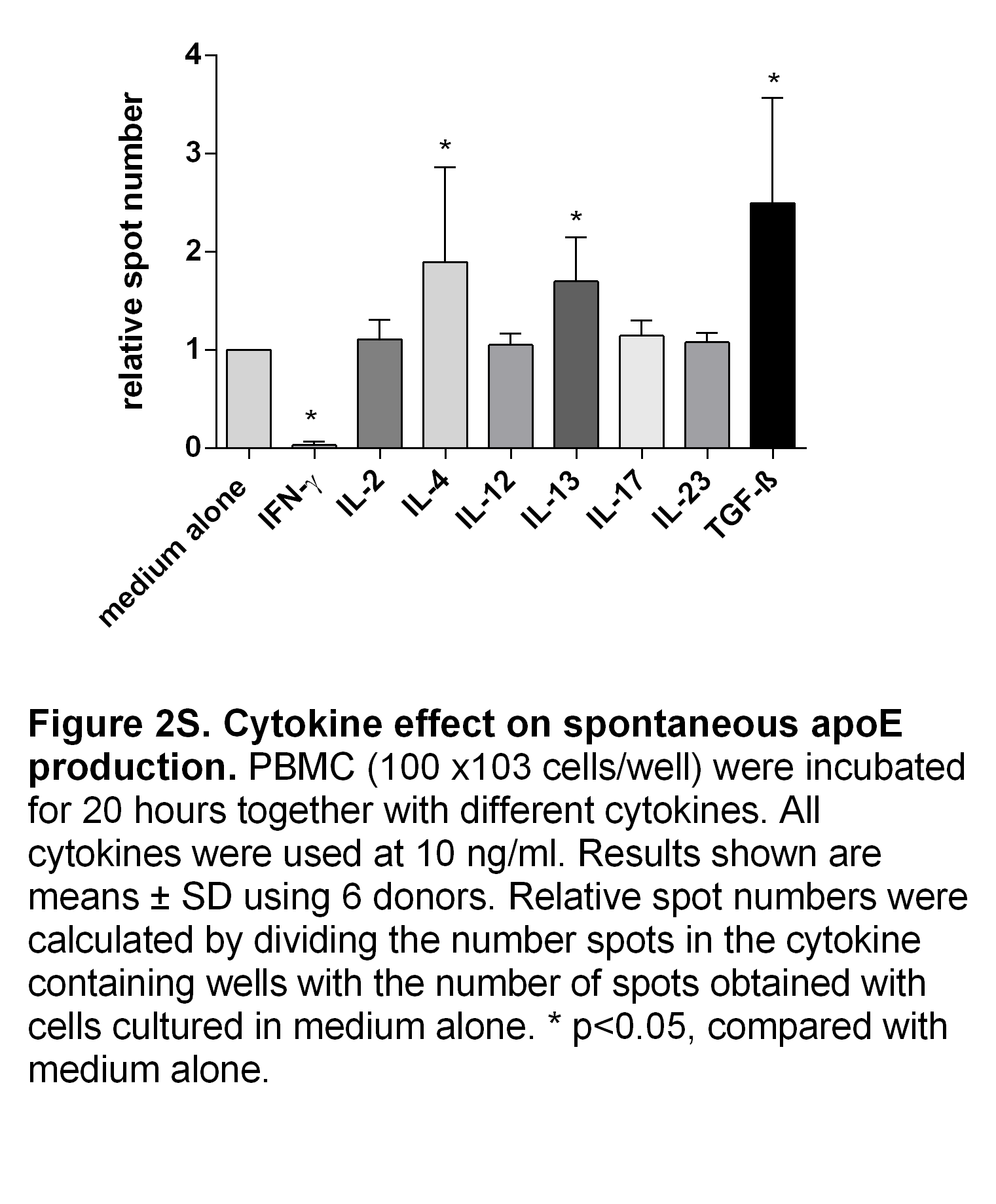

Supplement: Figure S2 — Cytokine effect on spontaneous apoE production. PBMC (100×103 cells/well) were incubated for 20 hours together with different cytokines. All cytokines were used at 10 ng/ml. Results shown are means ± SD using 6 donors. Relative spot numbers were calculated by dividing the number spots in the cytokine containing wells with the number of spots obtained with cells cultured in medium alone. * p<0.05, compared with medium alone. (TIF) [file pone.0079908.s002.tif]

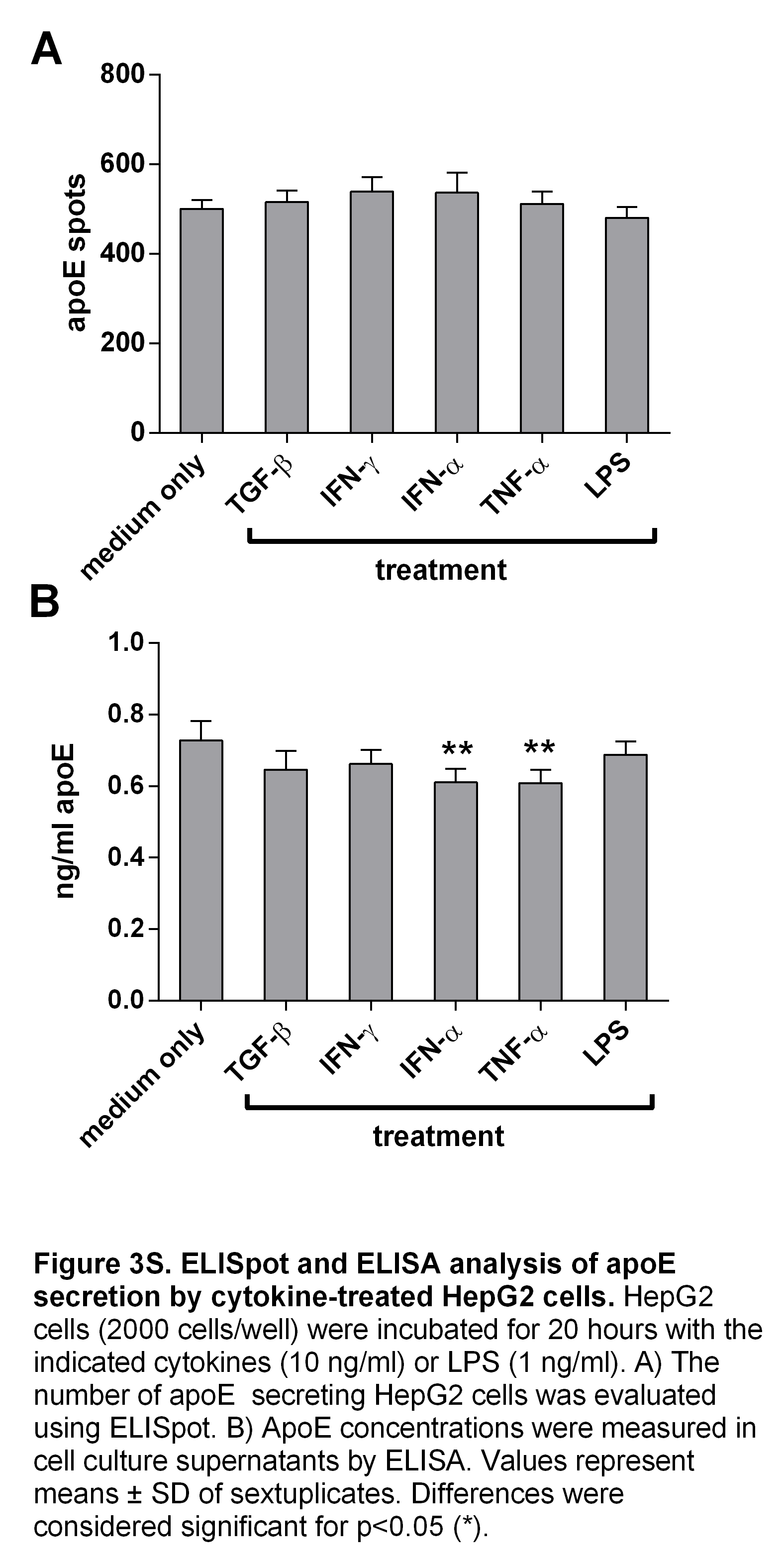

Supplement: Figure S3 — ELISpot and ELISA analysis of apoE secretion by cytokine-treated HepG2 cells. HepG2 cells (2000 cells/well) were incubated for 20 hours with the indicated cytokines (10 ng/ml) or LPS (1 ng/ml). A) The number of apoE secreting HepG2 cells was evaluated using ELISpot. B) ApoE concentrations were measured in cell culture supernatants by ELISA. Values represent means ± SD of sextuplicates. Differences were considered significant for p<0.05 (*). (TIF) [file pone.0079908.s003.tif]
